# Investigation and Management of Iron Deficiency Anemia in Non-pregnant Reproductive-Age Women: A Retrospective Analysis

**DOI:** 10.7759/cureus.107240

**Published:** 2026-04-17

**Authors:** Amal Peracha, Salman M Soomar, Shabeeha Rana

**Affiliations:** 1 Biomedical Science, King's College London, London, GBR; 2 Community Health Sciences, Agha Khan University, Karachi, PAK; 3 Research, King's College Hospital London, Dubai, ARE; 4 Hematology, King's College Hospital London, Dubai, ARE

**Keywords:** ferritin, heavy menstrual bleeding, intravenous iron, iron deficiency anemia, oral iron therapy, reproductive-age women

## Abstract

Background

Iron deficiency anemia (IDA) is the leading cause of anemia worldwide, disproportionately affecting women of reproductive age due to heavy menstrual bleeding (HMB). Although international guidelines emphasize baseline ferritin and hemoglobin measurement, treatment with oral or intravenous (IV) iron, and appropriate follow-up, gaps persist in practice. In the UAE, where prevalence remains moderate but clinically significant, evaluating adherence to evidence-based standards is critical. This study assessed compliance with international IDA guidelines at a private hospital in Dubai.

Methods

A retrospective chart review was conducted for non-pregnant women of reproductive age presenting with suspected IDA between January and June 2025. Data was collected on diagnostic testing, documentation of menstrual history, identification of underlying causes, treatment modality, and follow-up. Frequencies and percentages were calculated for categorical variables, while medians and interquartile ranges were reported for continuous data. Independent t-tests were performed to assess differences in hemoglobin and ferritin levels between patients with and without HMB, as well as to evaluate differences in treatment response between oral iron and IV iron. A t-test was applied, and a p-value ≤ 0.05 was considered statistically significant.

Results

Ninety-three patients were included. Hemoglobin was documented in 91.4% and ferritin in 86%. Only 41.9% met the diagnostic threshold of Hb <12 g/dL and ferritin <30 µg/L. Menstrual history was documented in 72%, but underlying causes such as HMB were addressed in 36.6%. Oral iron or Intravenous iron with appropriate justification was prescribed first-line in 86.0%, while IV iron use was justified in 80% of cases. Follow-up hemoglobin testing within the recommended timeframe was completed in only 15.1%.

Conclusion

High compliance was observed for initial investigations and oral iron prescribing, but shortfalls were identified in diagnostic accuracy, documentation of menstrual history, evaluation of underlying causes, and follow-up.

## Introduction

Iron deficiency (ID) is the most prevalent nutritional deficiency worldwide, affecting more than two billion people, with iron deficiency anemia (IDA) remaining the leading cause of anemia globally [1]. The World Health Organization (WHO) estimates that anemia affects approximately 30% of women aged 15-49 years, with iron deficiency accounting for the majority of cases [2]. Although iron homeostasis is tightly regulated in non-menstruating individuals, women of reproductive age are at increased risk due to ongoing physiological iron loss. As a result, this population bears a disproportionate burden of ID and IDA, leading to significant individual and health system consequences [1].

Heavy menstrual bleeding (HMB) represents one of the most important but under-recognized contributors to iron deficiency in reproductive-age women. Despite this, HMB remains substantially under-reported and under-investigated within routine clinical practice. Healthcare system data frequently estimate its prevalence at only 3%-5%, whereas survey-based studies suggest that up to half of women experience symptoms consistent with HMB. Many women normalize excessive bleeding and do not seek medical attention, while inconsistencies in clinical assessment further contribute to under-recognition [1,3,4]. Studies from Europe and North America demonstrate that even among women who present to healthcare services, evaluation and management are often suboptimal, resulting in delayed diagnosis and preventable morbidity [3,5,6].

The implications of this under-recognition extend beyond gynecological health. Evidence from a randomized trial in Finland showed that among women with HMB, 27% had established IDA and 90% had depleted iron stores, with more than half meeting criteria for severe iron deficiency [3,7]. Other studies report that up to two-thirds of women with HMB are anemic. These findings highlight the substantial contribution of HMB to iron depletion and emphasize the need to address the downstream effects of iron deficiency and anemia rather than focusing solely on etiological factors [3]. Untreated ID and IDA are associated with persistent fatigue, impaired concentration, reduced physical performance, and diminished quality of life. When prolonged, these effects can negatively influence occupational productivity, academic performance, and overall functional capacity [1].

Globally, the prevalence of IDA varies widely. Rates exceeding 40% are reported among women of reproductive age in South Asia and sub-Saharan Africa, while prevalence in high-income countries such as the United Kingdom and United States generally remains below 17% [8-12]. In the United Arab Emirates (UAE), anemia affects approximately 26.7% of women of reproductive age, a figure comparable to other Gulf Cooperation Council (GCC) countries including Qatar, Bahrain, and Kuwait [12-14]. Although lower than in low-income regions, this prevalence represents a clinically significant burden given the associated cognitive, physical, and quality-of-life impairments linked to untreated IDA.

Local evidence from the UAE further demonstrates the persistence of anemia despite relatively high awareness. A cross-sectional survey of female university students in Dubai reported anemia in 25% of participants. While overall knowledge of IDA symptoms, risk factors, and prevention strategies was high, misconceptions regarding diagnosis and management remained. Importantly, this level of awareness did not translate into improved prevalence rates, raising concerns regarding gaps between knowledge, clinical practice, and effective management [12,14]. This discrepancy underscores the need to examine how IDA is investigated and treated within healthcare settings rather than focusing solely on awareness initiatives.

Oral iron supplementation is widely recommended as first-line therapy due to its low cost and availability, with a typical 12-week course costing approximately £2.30. However, its effectiveness is frequently limited by gastrointestinal side effects, poor adherence, and variable absorption. Intravenous (IV) iron provides an alternative option, offering rapid iron repletion and improved tolerability, but at a substantially higher cost, estimated at approximately £1,400 over two admissions. While resource-intensive, IV iron may be cost-effective in selected patients by preventing prolonged symptoms, repeat treatment cycles, hospital admissions, and blood transfusions [15]. These considerations highlight the importance of appropriate treatment selection and follow-up in minimizing long-term health system burden [16,17].

International guidelines from the British Society for Hematology (BSH) and the National Institute for Health and Care Excellence (NICE) emphasize timely diagnosis, confirmation of iron deficiency through ferritin measurement, appropriate selection of oral or IV iron therapy, and post-treatment reassessment of hemoglobin and iron stores. Adherence to these evidence-based recommendations is critical for optimizing outcomes and avoiding recurrent or treatment-resistant anemia [18-22].

In the UAE, the moderate but persistent prevalence of IDA among women of reproductive age highlights the need to evaluate real-world adherence to these guidelines. Identifying gaps in diagnostic workup, treatment choice, and follow-up practices is essential for improving patient outcomes and reducing unnecessary healthcare utilization. Against this background, the present study evaluates compliance with evidence-based guidelines for the investigation and management of IDA in non-pregnant reproductive-age women at a private hospital in Dubai, with the aim of identifying opportunities for improving quality of care and clinical outcomes.

## Materials and methods

Study design

A retrospective study was conducted to assess compliance with guidelines for the investigation and management of IDA in non-pregnant reproductive-age women. The study period spanned from January to June 2025.

Study setting

The study was conducted at King’s College Hospital UAE, a multidisciplinary hospital that provides a wide range of specialist medical services, including Hematology. It is a Joint Commission International (JCI) Accredited hospital.

Study population and eligibility criteria

Female patients aged 15-45 years who were diagnosed with IDA during the six-month study period were included. Patients were excluded if they were pregnant or postmenopausal. Consent to participate was waived by the Institutional Ethics Review Committee, as it is a retrospective study.

Sampling method and sample size

A total of 93 patient records were included in this study. Records were initially identified using electronic health record (EHR) filters (female, aged 15-45, IDA diagnosis). Simple random sampling was used to enroll patients who met the inclusion criteria, as a line listing was available. The sample size of 93 reflects the total number of non‑pregnant women of reproductive age diagnosed with iron deficiency anemia who met the predefined inclusion criteria during the study period at the study site. As this was a retrospective audit of routine clinical practice, the sample size was determined by case availability rather than formal power calculation. 

Ethical approval and consent to participate

The research was carried out using the “Declaration of Helsinki.” Ethics approval was taken from the Ethics Review Committee of King’s College Hospital London, Dubai, UAE (KCH-REC-2025-082). Consent to participate was waived by the Institutional Ethics Review Committee, as it is a retrospective study.

Data collection and management

Data were collected retrospectively from the EHR using a structured data collection tool. The data collection tool was developed in‑house by the investigators. The tool was designed based on the study objectives and aligned with international guideline recommendations (BSH and NICE), incorporating parameters related to demographics, laboratory investigations, treatment modality, and follow‑up. Internal consistency of the tool was assessed using Cronbach’s alpha, which demonstrated acceptable reliability (α = 0.82). As this instrument was developed for a site‑specific retrospective audit, external validation was not performed. The outcome of the study was an evaluation of compliance with evidence-based guidelines in the investigation and management of iron deficiency anemia in non-pregnant reproductive-age women. Compliance was defined as documentation and management consistent with guideline recommendations, including appropriate diagnostic testing, treatment, and follow-up for both hemoglobin and ferretin. 

Operational definitions****

Anemia

Anemia is defined as a hemoglobin concentration of <12.0 g/dL in non‑pregnant women of reproductive age, in accordance with World Health Organization (WHO) criteria [12].

Heavy Menstrual Bleeding

HMB is identified based on clinical documentation in the electronic health record, as recorded by the treating physician. HMB was considered present if it was explicitly documented in the clinical notes. 

Statistical analysis

Descriptive statistics were used to summarize the data. Mean ± Standard Deviation (SD) was calculated for normally distributed quantitative continuous variables. Median and interquartile range (IQR) were calculated for skewed quantitative continuous variables. Normality was checked using the Shapiro-Wilk test for all quantitative variables. Categorical variables were presented as frequencies and percentages. Data visualization was performed using bar graphs using Microsoft Excel. In addition to descriptive statistics, inferential analyses were conducted to explore clinical correlations. The relationship between the presence of heavy menstrual bleeding (menorrhagia) and baseline hemoglobin and ferritin levels was assessed using an independent t-test. The significance of differences in treatment response between oral iron and IV iron was also evaluated. A p-value ≤ 0.05 was considered statistically significant.

## Results

The mean±SD age of patients was 35±7 years. Patients represented diverse nationalities, with the largest groups being from Europe & Central Asia (32.3%), South Asia (29%), and the Middle East & North Africa (22.6%). Of the 93 patients included in the study, baseline hemoglobin and ferritin were recorded in 91.4% (n = 85) and 86% (n=80), respectively. The median (IQR) baseline Hb was 8.9 (IQR: 7.8-10.2) g/dL, and the median (IQR) baseline ferritin was 12.75 (7-19.75) µg/L. Menstrual history was documented for 72% (n=67), and overall, 41.9% (n=39) reported menorrhagia (Table 1).

**Table 1:** Baseline demographic and clinical characteristics of patients (n= 93)

Variable	Value/n (%)
Age (years)	Mean 35 ± 7
15-19	3 (3.23%)
20-24	4 (4.30%)
25-29	14 (15.05%)
30-34	21 (22.58%)
35-39	21 (22.58%)
40-45	30 (32.26%)
Nationality	
South Asian	27 (29.03%)
Middle Eastern & North African (MENA)	21 (22.58%)
European & Central Asian	30 (32.26%)
American	9 (9.68%)
Sub-Saharan African	3 (3.23%)
Asia-Pacific (outside South Asia)	3 (3.23%)
Ferritin Tested	
Yes	80 (86.02%)
No	13 (13.98%)
Hemoglobin Tested	
Yes	85 (91.40%)
No	8 (8.60%)
Baseline Hemoglobin (g/dL) Median (IQR)	8.9 (7.8-10.2)
Baseline Ferritin (ng/mL) Median (IQR)	12.75 (7-19.75)
Menstrual History Recorded	67 (72.04%)
Heavy Menstrual Bleeding (HMB)	39 (41.94%)

With respect to treatment modality, oral iron or folic acid was prescribed in 47.3% of patients (n=44), while 48.4% (n=45) received IV iron therapy. A small proportion of patients either received no documented treatment or treatment details were unavailable.

Management of the underlying causes of anemia was documented in a subset of patients. Among those in whom underlying causes were addressed, 44.1% (n=15) were referred for gynecological evaluation, 23.5% (n=8) were prescribed tranexamic acid for HMB, and 32.4% (n=11) received other interventions.

Follow‑up laboratory monitoring was limited. Hemoglobin (Hb) was re‑measured in 38.7% of patients (n=36), while 61.3% (n=57) had no documented follow‑up Hb testing. Among those with repeat testing, the median hemoglobin level was 11.9 g/dL (IQR: 10.9-12.8), indicating only modest improvement following treatment.

Assessment of iron stores on follow‑up was also infrequent. Ferritin levels were rechecked in 30.1% of patients (n=28), whereas 69.9% (n=65) lacked documented follow‑up ferritin testing. Among patients with available data, follow‑up ferritin showed substantial replenishment of iron stores, with a median ferritin concentration of 204 ng/mL (IQR: 24.5-397). The wide interquartile range reflects marked variability in iron repletion, likely influenced by differences in treatment modality.

The interval between baseline testing and follow‑up assessment had a median duration of 36.5 days (IQR: 20-58.5) (Table 2).

**Table 2:** Treatment and follow-up characteristics of patients (n= 93)

Variable	Value/n (%)
Treatment Type	
Oral Iron/Folic acid	44 (47.31%)
IV Iron	45 (48.39%)
Addressed Underlying Causes	
Tranexamic Acid Prescribed	8 (23.53%)
Gynecologist Visit	15 (44.12%)
Others	11 (32.35%)
Follow-up Hb Done (Y/N)	
Yes	36 (38.71%)
No	57 (61.29%)
Hb Levels on Follow-up Median (IQR)	11.9 (12.8-10.9)
Follow-up Ferritin Done (Y/N)	
Yes	28 (30.12%)
No	65 (69.89%)
Ferritin Levels on Follow-up Median (IQR)	204 (397-24.5)
Days Between Testing Median (IQR)	36.5 (58.5-20)

**Figure 1:**
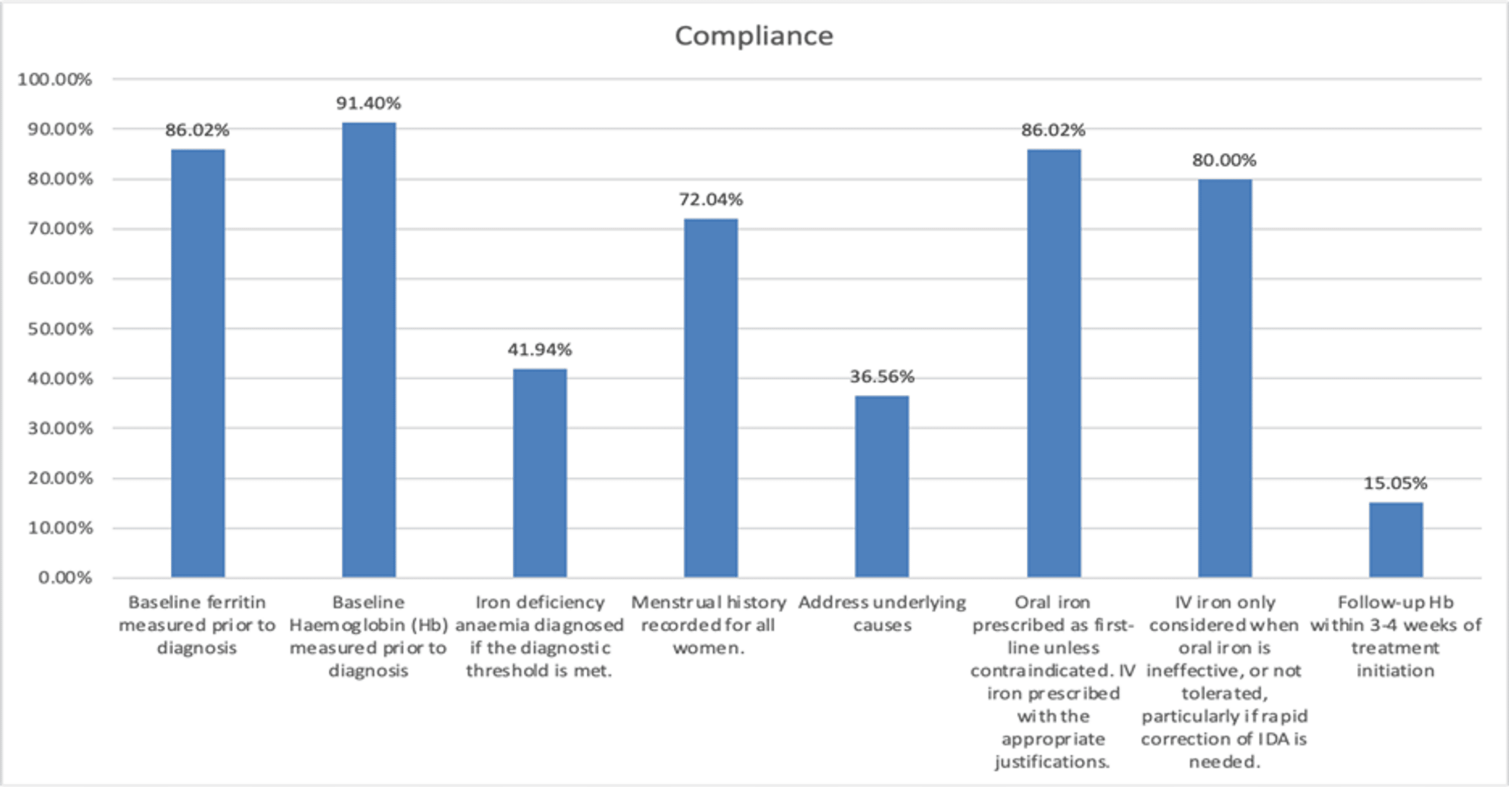
Compliance with guideline‑based standards for the diagnosis and management of IDA IDA: Iron deficiency anemia

Figure 1 summarizes compliance with predefined study standards. Baseline investigation showed good adherence, with hemoglobin measured in 91.4% (n=85) and ferritin in 86.0% (n=80) of patients. However, only 41.9% (n=39) fulfilled formal diagnostic criteria for iron deficiency anemia, indicating suboptimal diagnostic accuracy and potential over‑diagnosis. Menstrual history was documented in 70.97% (n=66) of cases.

Treatment adherence was variable. Oral iron was prescribed in 86.0% (n=80) of patients, while IV iron use was largely appropriate, with 80% (n=74) compliance with guideline indications. In contrast, underlying causes of anemia were addressed in only 36.6% (n=34) of patients. Follow‑up practices showed the poorest adherence, with hemoglobin reassessed within the recommended 3-4 weeks in only 15.1% (n=14) of cases.

The presence of menorrhagia was associated with significantly lower hemoglobin and ferritin levels. Patients with documented menorrhagia had a markedly lower median hemoglobin level compared with those without menorrhagia (8.2 g/dL vs. 12.0 g/dL; p=0.479). Similarly, median ferritin levels were lower among women with menorrhagia than those without (10.9 ng/mL vs. 12.2 ng/mL; p=0.423).

In contrast, treatment modality demonstrated a statistically significant effect on iron repletion. Patients treated with IV iron achieved a substantially greater median increase in ferritin levels compared with those receiving oral iron (402.7 µg/L vs. 26.1 µg/L; p <0.001) (Table 3).

**Table 3:** Association of baseline characteristics with clinical outcomes (n= 93) *t-test

Characteristics	Correlation	p-value*
Menorrhagia Present	Hemoglobin Levels (Median)	0.479
Yes	8.2	
No	12.0	
Menorrhagia Present	Ferritin Levels (Median)	0.423
Yes	10.9	
No	12.2	
Treatment Type	Change in Ferritin levels (Median)	<0.001
Oral Iron	26.1	
IV Iron	402.7	

## Discussion

This study demonstrates that baseline assessment was robust, with hemoglobin recorded in 91.4% and ferritin in 86% of patients, reflecting general awareness of the need for initial laboratory evaluation. However, only 41.9% of patients were formally diagnosed with IDA based on guideline-defined thresholds. Notably, several patients with values above the recommended ferritin cut-off were still labeled as anemic, reflecting a tendency toward over-diagnosis rather than under-recognition. This finding also highlights a potential limitation in current guidelines that rely on serum ferritin as the starting reference for diagnosing ID. In patients with inflammatory conditions, iron may be sequestered due to elevated hepcidin expression, restricting availability for erythropoiesis despite normal or even high ferritin levels. Under these circumstances, the standard threshold for ID (<30 μg/L) may not apply. Assessing transferrin saturation (TSAT) in patients with ferritin levels between 100 and 300 μg/L can improve diagnostic accuracy, better reflecting iron availability and allowing for timely initiation of treatment [23].

Menstrual history, a critical factor for identifying HMB as a contributor to iron loss, was documented in only 72% of cases, and underlying causes of anemia were addressed in just 36.6%. These findings suggest that while clinicians are performing baseline testing and initiating treatment, the evaluation of etiology and risk factors remains suboptimal, potentially leading to recurrent or unresolved ID.

Tables 2, 3 illustrate the impact of treatment on hemoglobin and ferritin levels, stratified by intervention type. IV iron produced a substantial increase in ferritin (mean Δ402.7 µg/L) compared with oral iron (mean Δ26.1 µg/L; p = 0.000529), emphasizing its rapid and reliable capacity to replenish iron stores. The wide interquartile range observed for post-treatment ferritin (24.5-397 µg/L) reflects heterogeneity in response, likely due to differences in baseline iron status, absorption, adherence, and the mode of administration. This variability underscores the importance of individualized monitoring and follow-up, as some patients achieved dramatic repletion with IV therapy while others had modest improvement, particularly with oral supplementation.

Hemoglobin levels demonstrated only modest improvement on follow‑up, despite substantial increases in ferritin levels. This is consistent with the known physiological lag between iron store repletion and subsequent hemoglobin synthesis. These findings highlight the limitations of using hemoglobin alone as a marker of treatment response and underscore the importance of ferritin monitoring to accurately assess iron repletion, particularly in clinical settings where rapid correction of ID is required.

When stratifying by clinical characteristics, patients with menorrhagia had a lower mean Hb (12.0 g/dL) compared with those without (13.2 g/dL; p = 0.479), and mean and median ferritin values were 30.5 µg/L and 10.9 µg/L versus 19.5 µg/L and 12.2 µg/L, respectively (p = 0.423). Although the differences were not statistically significant, the discrepancy between mean and median ferritin in both groups indicates a skewed distribution, with a subset of patients showing disproportionately high ferritin levels, likely reflecting prior IV iron therapy or individual variability in iron metabolism. Previous literature consistently associates menorrhagia with lower iron stores and increased risk of IDA; thus, the absence of statistical significance in this cohort is likely explained by small sample size, heterogeneity of treatment modalities, incomplete follow-up, and biological variability, rather than a true lack of effect. The median values, being lower than the means in both groups, highlight that a typical patient with menorrhagia may have substantially lower iron stores than suggested by mean values, reinforcing the clinical relevance of evaluating central tendency beyond simple means.

Our findings of under-recognition of IDA and inconsistent documentation of menstrual history highlight a critical gap between clinical practice and international recommendations. The International Federation of Gynecology and Obstetrics (FIGO) advises that all reproductive-aged girls and women should be routinely screened for ID from menarche onward, ideally through serum ferritin measurement and, in cases where chronic inflammation is suspected, TSAT. Furthermore, FIGO emphasizes that when ID or IDA are identified in nonpregnant women of reproductive age, heavy menstrual bleeding should be actively suspected, investigated, and managed [24]. Improved adherence to these guidelines could prevent prolonged iron loss, reduce the need for repeated replacement therapy, and ultimately optimize both patient outcomes and healthcare resource utilization.

The findings demonstrate that while baseline testing and prescription practices are generally strong, critical gaps exist in systematic diagnosis, investigation of underlying causes, and follow-up monitoring. These gaps are clinically significant: undiagnosed or inadequately treated IDA may persist, leading to fatigue, impaired cognitive function, and decreased quality of life. The substantial efficacy of IV iron suggests it should be considered for patients with poor oral tolerance, significant ongoing blood loss, or urgent clinical needs, but cost, access, and clinician awareness remain barriers. The wide variability in ferritin response further emphasizes the need for personalized follow-up, particularly for high-risk groups such as women with menorrhagia.

Strengths and limitations

This study is one of the few to investigate IDA in reproductive-age women within the UAE, contributing valuable insights into regional clinical practice. The inclusion of both hematological outcomes and documentation practices (such as menstrual history) allows for a comprehensive assessment of diagnostic and management gaps. Additionally, the study’s focus on both oral and IV iron treatment responses provides practical implications for optimizing therapy selection.

However, several limitations should be acknowledged. The study was conducted in a single center, which may limit the generalizability of findings to broader populations. As a retrospective chart review, there is a risk of information bias due to incomplete or inconsistent documentation. Selection bias is also possible, given the reliance on available hospital records rather than systematic recruitment. The data does not report TSAT, which prevents evaluating iron availability in patients where ferritin may be unreliable due to inflammation. The underlying causes of IDA were not consistently documented in the electronic health records and therefore could not be systematically analyzed. As a result, the study was unable to stratify findings based on etiology, which may limit interpretation of treatment choices and follow‑up practices.

## Conclusions

This study highlights key gaps in the recognition and management of IDA in reproductive-age women. While iron studies were frequently available, only 41.9% of patients were formally diagnosed according to guideline-defined thresholds. Documentation of menstrual history occurred in 72% of patients, limiting systematic identification of HMB as a primary etiology. Treatment responses showed improvement in hematological parameters with both oral and IV iron, though variability was evident, particularly among patients with menorrhagia. These findings underscore the importance of adhering to evidence-based diagnostic thresholds, comprehensive assessment of underlying causes, and structured follow-up to ensure timely identification and effective management of IDA in this population. Greater clinical awareness, alongside structured protocols for screening and follow-up, is essential to optimize outcomes and reduce the long-term healthcare burden of untreated or recurrent IDA in this population.
